# Impaired VEGF-A-Mediated Neurovascular Crosstalk Induced by SARS-CoV-2 Spike Protein: A Potential Hypothesis Explaining Long COVID-19 Symptoms and COVID-19 Vaccine Side Effects?

**DOI:** 10.3390/microorganisms10122452

**Published:** 2022-12-12

**Authors:** Rossella Talotta

**Affiliations:** Rheumatology Unit, Department of Clinical and Experimental Medicine, University of Messina, University Hospital “G. Martino”, 98124 Messina, Italy; rtalotta@unime.it

**Keywords:** SARS-CoV-2, long COVID-19, post-COVID-19, COVID-19 vaccine, VEGF-A, spike protein, NRP-1, small fiber neuropathy

## Abstract

Long coronavirus disease-19 (COVID-19) is a newly discovered syndrome characterized by multiple organ manifestations that persist for weeks to months, following the recovery from acute disease. Occasionally, neurological and cardiovascular side effects mimicking long COVID-19 have been reported in recipients of COVID-19 vaccines. Hypothetically, the clinical similarity could be due to a shared pathogenic role of the severe acute respiratory syndrome coronavirus-2 (SARS-CoV-2) spike (S) protein produced by the virus or used for immunization. The S protein can bind to neuropilin (NRP)-1, which normally functions as a coreceptor for the vascular endothelial growth factor (VEGF)-A. By antagonizing the docking of VEGF-A to NRP-1, the S protein could disrupt physiological pathways involved in angiogenesis and nociception. One consequence could be the increase in unbound forms of VEGF-A that could bind to other receptors. SARS-CoV-2-infected individuals may exhibit increased plasma levels of VEGF-A during both acute illness and convalescence, which could be responsible for diffuse microvascular and neurological damage. A few studies suggest that serum VEGF-A may also be a potential biomarker for long COVID-19, whereas evidence for COVID-19 vaccines is lacking and merits further investigation.

## 1. Introduction

A large body of research studies and publications has characterized the last two years. These studies have helped to better understand the pathogenesis, risk factors, clinical course, prevention, and treatment of coronavirus disease-19 (COVID-19). COVID-19 is an infectious disease caused by severe acute respiratory syndrome coronavirus 2 (SARS-CoV-2), which first broke out in Wuhan, China, in December 2019 [1]. The rapid spread of the infection to other countries prompted the World Health Organization (WHO) to declare a Public Health Emergency of International Concern in January 2020, and to designate the outbreak as a pandemic in March 2020 [2]. By November 2022, more than 260 million cases had been confirmed in European countries and more than 2 million people had died from the disease [2], mainly due to lung, heart, kidney, and liver failure [3,4]. In the meantime, tremendous efforts have been made to find effective drugs and vaccines to contain the spread of the infection around the world and treat its most severe forms.

SARS-CoV-2 is a single-stranded RNA-enveloped virus that belongs to the betacoronavirus family. Its genome encodes structural and nonstructural proteins, of which the spike (S) protein is critically involved in cell infectivity. The S proteins are located on the surface of SARS-CoV-2 and promote viral entry by interacting with the host cell receptor angiotensin-converting enzyme 2 (ACE2) and transmembrane serine protease 2 (TMPRSS2) through their receptor-binding domain (RBD) [5]. ACE2 is abundantly expressed in cells of the respiratory and gastrointestinal tracts as well as the heart and kidneys, which explains the pleotropic manifestations of COVID-19 [6]. In addition to ACE2, the SARS-CoV-2 S protein can also engage the receptor CD147 and infect cells that lack ACE2, such as immune cells [7]. Recently, it has been shown that SARS-CoV-2 uptake can occur via a different pathway that exploits S protein binding to neuropilin (NRP)-1, which normally binds vascular endothelial growth factor (VEGF)-A, collapsin/semaphorin proteins, and furin-cleaved substrates in nerves and vessels [8,9].

Mutations in the S protein have led to the emergence of novel variants of concern (VOC), endowed with higher infectivity and capable of evading the immune response and challenging the efficacy of vaccines, monoclonal antibodies, and convalescent sera [10]. The original Alpha B.1.1.7 variant has been displaced by the Delta variant and more recently by the Omicron variants, which are characterized by increased transmissibility due to more than 30 mutations in the S protein [11]. The recently discovered Omicron BA.2.75 subvariant, also known as the “Centaurus subvariant”, has raised the attention of scientists due to its increased infectivity, which appears to depend on a >3000-fold affinity for ACE2 compared to the Alpha B.1.1.7 variant [12]. Although responsible for successive COVID-19 waves, epidemiological studies have shown that new variants are associated with faster recovery and lower rates of hospitalization, even in unvaccinated individuals, than the original Wuhan strain [13,14,15].

The emergence of milder variants, the formulation of more effective therapeutic strategies, and the worldwide vaccination campaign have reduced the risk of severe disease. However, the focus has shifted in cases of persistent disabling symptoms that possibly occur in a fraction of recovered individuals as well as in some recipients of COVID-19 vaccines. With the weakening of political restrictions and higher virus circulation, as well as the globalization of vaccination programs, these events are expected to be predominant among future COVID-19-related concerns.

The long-term effects of COVID-19, termed long COVID-19 or post-COVID-19, can affect physical and mental health, impair work productivity, and thus require immediate identification and appropriate interventions [16]. Cases of long COVID-19 have been reported in a proportion of individuals infected with both Omicron and earlier variants, although the likelihood of experiencing long COVID-19 symptoms appears to be lower in patients infected with the Omicron strains [17]. Interestingly, some of the manifestations reported during long COVID-19, such as fatigue, myalgia, palpitations, headaches, and dizziness, may overlap with the side effects of COVID-19 vaccines [18,19,20,21,22,23,24,25,26]. Such manifestations are predominantly neurological and cardiovascular in nature and could hypothetically be attributed to dysfunctional mechanisms triggered by the S protein produced during infection or used for immunization, which could bind to NRP-1 in the neurovascular district. By antagonizing the docking of VEGF-A to NRP-1, it may be presumed that the S protein could potentially disrupt physiological pathways involved in angiogenesis and nociception [8,27,28,29], contributing to the neurological and cardiovascular manifestations seen in SARS-CoV-2-infected individuals as well as in some COVID-19 vaccine recipients. Research has already shed light on the possibility of a “spike intoxication”, also referred to as the “spike effect”, in terms of saturation and subsequent impairment of ACE2 receptor function [30,31,32,33,34]. Although current evidence in support is scarce, it may be hypothesized that such a mechanism could also occur with respect to NRP-1 and perhaps contribute to the development of neurological and cardiovascular symptoms of long COVID-19 or some side effects of COVID-19 vaccines. Therefore, the aim of this review is to summarize the evidence supporting this hypothesis and to discuss potential pitfalls by performing a literature search in PubMed and Google Scholar databases using combinations of the following terms: “SARS-CoV-2”; “COVID-19”; “long COVID-19”; “post-COVID-19” “COVID-19 vaccine”; “VEGF-A”; “neuropilin-1”. The results from the selected studies will be discussed in the following sections.

## 2. The NRP-1/VEGF-A Complex in Health and Disease

### 2.1. The Family of NRPs

The family of NRPs includes NRP-1 and NRP-2, which are transmembrane non-tyrosine kinase glycoproteins with extracellular semaphorin- and VEGF-binding sites [35]. NRP-1 and NRP-2 have up to 55% of homology [36] and are expressed in a variety of cells such as endothelial cells, neurons, glial cells, and immune cells, being therefore involved in several physiological processes [35,37]. Both receptors contain three extracellular domains that can interact with class three semaphorins, growth factors, and membrane receptors [36,38]. Alternative splicing can give rise to different NRP-1 and NRP-2 isoforms, some of which are soluble and function as decoy receptors [36].

NRP-1 was originally discovered in the nervous system, where it may be involved in neurogenesis and axonal pruning by binding to collapsin/semaphorin ligands [9]. In addition, NRP-1 may act as a coreceptor for VEGF-A in the endothelium, controlling vascular remodeling and angiogenesis [39]. Other interactions with growth factors, including transforming growth factor (TGF)-β, fibroblast growth factor (FGF), hepatocyte growth factor (HGF), platelet-derived growth factor (PDGF), or activated tyrosine kinase receptors may explain the further roles that NRP-1 plays in cell proliferation, migration, survival, and invasion during embryogenesis or carcinogenesis [37,38]. Moreover, NRP-1 appears to be involved in immunologic synapse formation, B-cell differentiation, and immunotolerance [36].

In contrast, the physiological functions of NRP-2 are less well described. This receptor can bind to distinct semaphorin and VEGF ligands and guide the development of the lymphatic system during embryogenesis [40]. In addition, NRP-2 is relatively abundant in immune cells, where it may control important functions such as chemotaxis, phagocytosis, or antigen presentation [41].

### 2.2. The Family of VEGFs

Eight members make up the family of VEGFs, namely VEGF-A, VEGF-B, VEGF-C, VEGF-D, VEGF-E, VEGF-F, placental growth factor (PlGF), and endocrine gland-derived (EG)-VEGF [42]. Like NRPs, VEGFs are produced by different cell types, including endothelial cells, neurons, and glial cells. Once secreted, they can bind to the tyrosine kinase VEGF-receptor (VEGF-R) 1, VEGF-R2, and VEGF-R3, whose activation triggers various intracellular signaling cascades. VEGF-A can be spliced into the VEGFxxxa isoforms VEGF165, VEGF121, and VEGF206, which differ in terms of heparin and heparan sulphate proteoglycan-binding activity, and VEGFxxxb isoforms having opposite effects [42,43,44]. VEGF-A, and more specifically its variant VEGF165, is the major family member involved in angiogenesis [37]. Its secretion is enhanced during hypoxia, inflammation or mitochondrial stress through several pathways mediated by hypoxia-inducible factors (HIFs), peroxisome proliferator-activated receptor-γ coactivator (PGC)-1, tumor necrosis factor (TNF)-α, and nuclear factor kappa-light-chain-enhancer of activated B cells (NF-kB) [42]. Together with VEGF-B, VEGF-A can bind to VEGF-R1 and VEGF-R2 in endothelium, while VEGF-C and VEGF-D interact with VEGF-R3 in lymphatic vessels [35]. Acting as coreceptors, NRP-1 and NRP-2 enhance the binding of VEGF-A to VEGF-R2 in blood vessels and that of VEGF-C to VEGF-R3 in lymphatic vessels, respectively [35,45]. Interestingly, a quantitative FRET analysis showed that NRP-1 may interact with VEGF-R2 in the plasma membrane regardless of the presence of VEGF165, and that both VEGF165 and VEGF-R2 binding may rearrange NRP-1 molecular structure, preventing homo-oligomerization [46].

VEGF-R1 is also expressed on the membrane of monocytes and macrophages and its stimulation can lead to inflammation [47]. Remarkably, NRP-1 was found to reversibly associate with VEGFR-1 and impair the binding of VEGF-A165, thereby preventing the downstream signaling activity of VEGF-R1 [48]. In the endothelium, VEGF-R1 has a higher binding affinity for VEGF-A than VEGF-R2, but its kinase activity is tenfold weaker, suggesting that VEGF-R1 may function as a control receptor during angiogenesis [47]. Instead, the stimulation of the endothelial VEGF-R2 promotes vascular permeability and new vessel formation through the activation of protein kinase C (PKC), mitogen-activated protein kinase (MAPK), and phosphatidylinositol 3-kinase (PI3K)/protein kinase B (AKT) signaling pathways [49,50].

Furthermore, the activation of VEGF-R2 in the nervous system can lead to the proliferation, migration, and survival of neurons, astrocytes, microglia, Schwann cells, and stem cells [50]. In addition to neurogenesis, the activation of the NRP-1/VEGF-A–VEGF-R2 pathway in myelinated A fibers and unmyelinated C fibers can cause hyperalgesia and allodynia [51].

### 2.3. Physiopathological Crosstalk between NRPs and VEGFs

**Figure 1:**
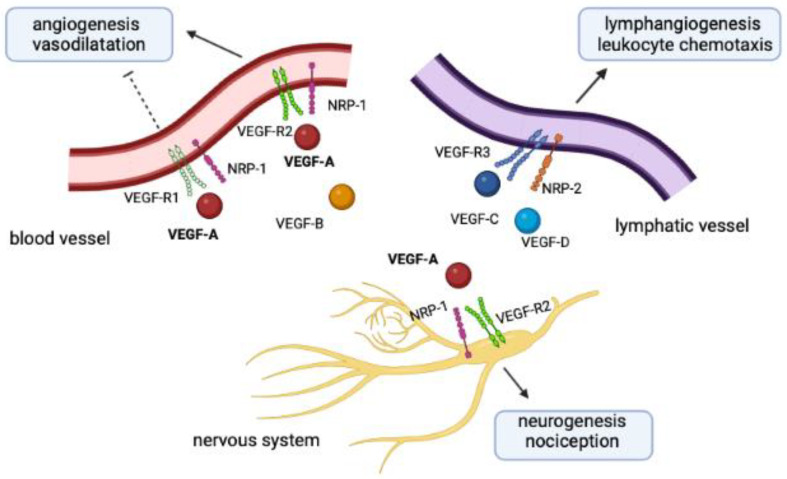
Physiological pathways directed by the NRP/VEGF complex in vessels and nervous system. Blood vessels, lymphatic vessels, and the nervous system express VEGF-Rs and NRPs, both of which function as receptors for VEGFs. Angiogenesis and vasodilation are the final effects of the interaction between VEGF-A/VEGF-B and the receptor complex VEGF-R1/VEGF-R2–NRP-1 in blood vessels. VEGF-C and VEGF-D instead bind to the VEGF-R3–NRP-2 receptor complex in lymphatic vessels, which may lead to lymphangiogenesis and chemotaxis of leukocytes. Finally, VEGF-A can control neurogenesis and nociception by binding to the receptor complex VEGF-R2–NRP-1 in the nervous system. Nerve fibers may in turn release VEGF-A, which has paracrine effects on receptors expressed on patterned vessels. Abbreviations: NRP: neuropilin; VEGF: vascular endothelial growth factor; VEGF-R: vascular endothelial growth factor receptor. Created with BioRender.com.

The interplay of NRPs, VEGF-Rs, and VEGFs reflects the close interconnection between nerves and vessels from both an anatomic and functional perspective, as seen in Figure 1. Arteries are indeed patterned with peripheral nerves and the latter play a crucial role in the alignment and differentiation of nerves and vessels during arteriogenesis. A preclinical study on nerve-specific Cre lines has shown that VEGF-A can be produced locally by sensory neurons, motor neurons and Schwann cells. Once released near blood vessels, VEGF-A may upregulate the endothelial expression of NRP-1 and further promote sensitivity to VEGF-A [52].

In humans, the NRP-1/VEGF-A complex has been extensively studied in cancer, due to its role in tumor progression and invasiveness [53,54,55,56,57]. Regardless of tumor type, malignant cells overexpress NRP-1 and are thus more sensitive to the mitogenic effects of VEGF and other mediators [58]. Consistent with this view, the anti-VEGF-A monoclonal antibody bevacizumab has been approved for the treatment of several cancers, including metastatic colorectal cancer, metastatic breast cancer, and ovarian cancer [59]. Other compounds that can antagonize NRP-1 have shown promise in preclinical cancer studies [37].

Preclinical evidence also shows that NRP-1 may exert cardioprotective effects. Using mouse models, Wang et al. found that the deletion of NRP-1 in cardiac cells and vascular smooth muscle cells resulted in cardiac hypertrophy and mitochondrial stress, and that it reduced cardiorespiratory fitness in the animals [60]. These effects were attributed to a dysregulated signaling pathway involving TGF-β and PGC-1, but not VEGF-A. In another experiment using mice and cell models, the same group of authors observed that NRP-1 may downregulate the expression of asymmetric dimethylarginine and reduce the sensitivity of endothelial cells to low-dose angiotensin II, with significant effects on blood pressure control [61].

Regarding the role of the NRP-1/VEGF-A system in neurological diseases, some reports have shown the aberrant expression of semaphorin-3A and semaphorin-7A in multiple sclerosis (MS) lesions and the association with impaired differentiation capacity of oligodendrocyte precursors [62]. Conversely, the expression of NRP-1 in microglia and CD4+ T cells appears to improve the outcome in models of experimental autoimmune encephalomyelitis [63,64]. Impaired NRP-1 activity may also underlie dysfunctional neurological symptoms. Indeed, the results of a genotyping study of more than 200 symptomatic women revealed a significant association between a polymorphic variant near the NRP-1 gene and the risk of developing menstrual migraine [65].

**Figure 2:**
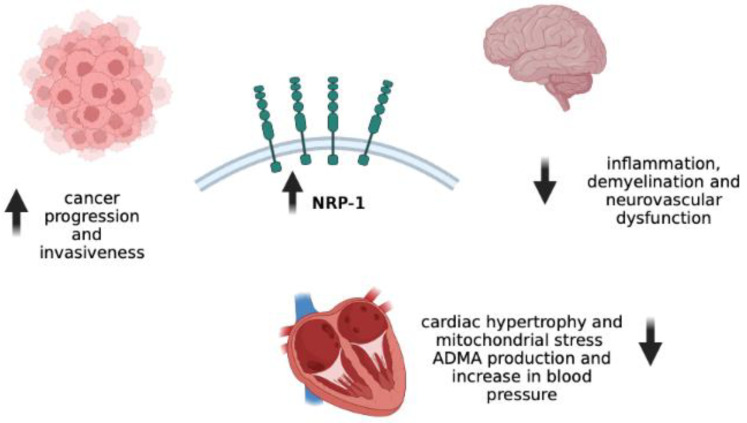
The potential contribution of NRP-1 to human disease. Overexpression of NRP-1 in different clinical contexts may play either a protective or a pathogenic role. In cancer, the receptor may contribute to malignant cell proliferation and metastasis. Conversely, when upregulated in the nervous system, NRP-1 would prevent neuronal dysfunction, inflammation, and demyelination. When expressed on cardiomyocytes and vascular smooth muscle cells, NRP-1 could counteract cardiac hypertrophy and regulate vascular tone. Abbreviations: ADMA: asymmetric dimethylarginine; NRP-1: neuropilin-1. Created with BioRender.com.

The clinical effects of NRP-1 overexpression are shown in Figure 2.

On the other hand, VEGF-A is typically overproduced in patients with polyneuropathy, organomegaly, endocrinopathy, M-protein, skin changes (POEMS) syndrome, a rare plasma cell disorder of unknown etiology, also being correlated with disease activity [66,67]. In these individuals, the increase in VEGF-A may be at the basis of manifestations such as extravascular volume overload, papilledema, thrombophilia, and abnormal pulmonary function tests [67]. Interestingly, POEMS polyneuropathy is usually demyelinating and affects both the sensitivity and motor functions of the extremities.

Elevated levels of circulating VEGF-A are also a marker for the remitting seronegative symmetrical synovitis with pitting edema (RS3PE) syndrome, a rheumatic disease characterized by symmetrical synovitis with increased vascular permeability [68]. Since VEGF-A is closely associated with systemic inflammation, it can also be overproduced in patients with other rheumatic diseases, including rheumatoid arthritis and polymyalgia rheumatica [69,70,71].

## 3. The NRP-1/VEGF-A Pathway during Human Viral Infections

Evidence suggests that the NRP-1/VEGF-A pathway may participate in the pathogenesis of viral diseases other than COVID-19. To date, an NRP-1-dependent entry route has been solely described for human T-cell lymphotropic virus type 1 (HTLV-1). HTLV-1 is a human retrovirus able to infect CD4+ and CD8+ T lymphocytes, monocytes/macrophages, and dendritic cells. The infection may result in lymphoproliferative and inflammatory disorders or neuromyelopathies. In vitro experiments have shown that the surface subunit of the HTLV-1 env protein may mimic human VEGF165 and thus promote the viral entry in dendritic cells or CD4+ T lymphocytes expressing NRP-1 on the plasma membrane [72]. Similarly, the expression of NRP-1 on human cerebral endothelial cells may enable HTLV-1 infection and alter the blood–brain barrier, perhaps explaining the development of neurological manifestations such as HTLV-1-associated myelopathy/tropical spastic paraparesis [73].

Changes in the levels of VEGF have been described during both bacterial and viral infections [74,75]. Human viruses such as herpesviruses, hepatitis viruses, or human papillomavirus may upregulate the expression of VEGF directly or indirectly through many mechanisms involving HIF-1α, cyclooxygenase 2 (COX2), activator protein-1 (AP-1), NF-kB, interleukin (IL)-6 or, as in the case of Orf viruses, through the synthesis of viral VEGF homologs [76]. The subsequent effects on angiogenesis and vascular permeability may help oncogenic and non-oncogenic viruses spread to other tissues or induce tumor progression. Therefore, the overproduction of VEGF appears as a common tract of infections or inflammation, being thus not specific to COVID-19.

Concerning viral respiratory infections, there are several reports showing elevated levels of VEGF in biological samples of patients with influenza and respiratory syncytial virus (RSV) infection, in whom VEGF also appeared as a marker of disease severity [75,77,78,79,80], as shown in Table 1. Importantly, the results of these studies indicate that the release of VEGF in response to viral infection would not occur as an isolated event, rather being inscribed into a molecular network characterized by concomitant increases of proinflammatory systemic cytokines or chemokines such as IL-6, IL-8, and monocyte chemoattractant protein-1 (MCP-1), or unbalances in leukocyte subsets. These clinical observations have been confirmed by laboratory studies revealing that VEGF may be released by primary bronchial or pulmonary epithelial cells and mast cells upon respiratory viral infections in the attempt to potentiate the type I and II interferon (IFN) response [74,81,82,83].

Conversely, there are no data concerning a dysregulation in the VEGF pathway during infections sustained by human coronaviruses other than SARS-CoV-2, such as human coronavirus (HCoV)-NL63, HCoV-HKU1, HCoV-229E, HCoV-OC43, SARS-CoV, or Middle East respiratory syndrome coronavirus (MERS-CoV). The alphacoronaviruses HCoV-NL63/HCoV-229E and the betacoronaviruses HCoV-HKU1/HCoV-OC43 are widespread causative agents of the common cold and other mild-to-moderate respiratory infections [84]. SARS-CoV was instead responsible for the outbreak of a severe form of SARS in China in the years 2002–2003, characterized by a high mortality rate but also a limited transmission of the infection [85]. Finally, a MERS-CoV infection developed in Middle Eastern countries in 2012 and was characterized by a highly aggressive respiratory disease with a mortality rate peaking up to 50% of infected individuals [85].

SARS-CoV S protein shares 76% identity and 86% similarity with the SARS-CoV-2 S protein, and both can interact with ACE2 as the main receptor [86,87]. However, identity decreases to 74% when considering the RBD that mediates receptor attachment, and to 61% when considering the whole S1 subdomain [88]. These differences may account for the four-fold increased affinity for ACE2 displayed by the SARS-CoV-2 RBD compared with SARS-CoV. Conversely, the RBDs of MERS and other HCoVs display little sequence similarity with the RBD of SARS-CoVs, which may account for distinct receptor affinities [89,90,91]. To date, it is unknown whether the S protein of coronaviruses other than SARS-CoV-2 may bind to NRP-1 or disrupt VEGF-related pathways [91,92].

## 4. Disruption of the NRP-1/VEGF-A Pathway by SARS-CoV-2 in Acute COVID-19

COVID-19 is a polyhedral disease characterized by a range of manifestations of varying intensity [93]. Despite being less common with the Omicron variant infection, reported symptoms usually include fever, fatigue, myalgia, cough, headache, hypo-anosmia, and hypo-ageusia [94,95]. The prognosis is generally more severe in the case of viral pneumonia with subsequent development of hypoxemic respiratory failure and acute respiratory distress syndrome (ARDS) [96]. Occasionally, patients may suffer from cardiac symptoms (arrhythmia, myocarditis, acute coronary syndrome, and heart failure), neuropsychiatric manifestations (stroke, seizure, encephalopathy, demyelinating disorders, or psychosis), and thromboembolic events [96].

A growing body of evidence suggests that COVID-19 is due to a profound alteration of the endothelium triggered directly by SARS-CoV-2 or indirectly by the increase in proinflammatory cytokines [97]. This view is supported by manifestations consistent with diffuse or organ-specific endotheliitis, altered nailfold capillaroscopy patterns in COVID-19 patients, and increases in markers of endothelial injury in blood samples from infected individuals, such as circulating endothelial cells (CECs), von Willebrand factor, soluble intercellular adhesion molecule-1 (sICAM-1), and angiopoietin-2 (Ang-2) [97,98,99,100]. In addition, COVID-19 patients exhibit increased plasma levels of VEGF-A during both acute disease and convalescence, possibly reflecting diffuse microvascular injury [101,102,103]. Endothelial dysfunction leading to local tissue hypoxia could be another stimulus for VEGF-A secretion, triggering a self-perpetuating mechanism [42]. The increase in VEGF-A and other angiogenic factors may impair pericyte function and lead to intussusceptive angiogenesis in the lungs of infected individuals, contributing to endothelial hyperpermeability and massive interstitial edema [104,105,106].

Recent studies have downplayed the role of ACE2 in enabling SARS-CoV-2 entry into endothelial cells and nervous cells [97,107]. Although ACE2 can be detected in human endothelium, mature human cortex and olfactory cells, its expression is very low and probably insufficient to cause direct infection. Therefore, endothelial and neurological damages that occur during COVID-19 may depend on alternative mechanisms, including the exploitation of various cell receptors by the virus [108]. A number of experiments have shown that SARS-CoV-2 may be capable of interacting with the membrane NRP-1 via its S protein and subsequently being internalized through this pathway [27,109,110,111,112]. Specifically, the B1 domain of NRP-1 has been predicted to interact with furin-cleaved ligands that contain a shared motif with arginine amino acid residues, also known as R/KXXR sequence, that is common to VEGF members [113]. A computational study revealed that a polybasic sequence in the C-terminus of the furin-cleaved SARS-CoV-2 S1 subdomain may also associate with NRP-1 in the B1 domain, potentially displacing the VEGF ligand from the binding site [114], as shown in Table 2. However, this kind of binding appeared to be flexible and transient, and thus less effective than other molecular conformational interactions.

The emergence of new SARS-CoV-2 variants may indeed affect the binding affinity of the S protein to its receptors, modulating viral infectivity; however, data concerning changes in the avidity for NRP-1 receptors are unclear [115,116].

Given the abundant expression of NRP-1 in the endothelium as well as in the nervous system, olfactory epithelium, and respiratory system, this alternative mechanism could explain the COVID-19 endotheliitis and other complications such as anosmia, dizziness, and headache [8,9,117,118]. Interestingly, a recent experiment on induced pluripotent stem cell (iPSC)-derived brain organoids and primary human astrocytes from the cerebral cortex found that SARS-CoV-2 may invade astrocytes by using NRP-1 as the primary receptor [116]. Furthermore, as shown by Moutal et al. in a preclinical assay using rat models and spinal ganglion neurons [27], the binding of the S protein to NRP-1 may also impede nociception and explain a more rapid viral transmission in asymptomatic individuals. Moreover, the release of proinflammatory cytokines during acute illness may upregulate the expression of NRP-1 in the cardiomyocytes of SARS-CoV-2-infected patients, facilitating viral infectivity and cellular injury [119].

The inflammatory background associated with acute COVID-19 may also promote the activation of the VEGF-A/VEGF-R2 pathway and induce angiogenesis, increased vascular permeability, nitric oxide production, and disruption of endothelial cell junctions [97]. The excess of VEGF-A may then promote cardiac edema, inflammation, and remodeling of myocyte interstitial spaces, eventually leading to cardiac arrhythmias, as shown in a study of animal models [120]. Finally, VEGF-A may affect nociception and contribute to COVID-19 neuropathy [27].

As previously mentioned, the upregulation in the VEGF pathway is not exclusive to COVID-19, also being observed during other viral infections. Different trends, however, emerged when comparing VEGF expression during SARS-CoV-2 infection and infections sustained by other respiratory viruses. In a prospective cohort study evaluating the immune profile of moderate-to-severe COVID-19 and pandemic influenza A(H1N1) patients, a higher serum expression of VEGF was found in the former group compared to the latter [121]. Similarly, a Chinese study conducted on hospitalized COVID-19 children, children with acute respiratory tract infections caused by RSV, influenza virus, and adenovirus, and 20 matched healthy controls reported a panel of dysregulated cytokines in all the diseases, among which VEGF was the only one significantly associated with COVID-19 [122].

Based on these findings, VEGF-A has been considered a potential biomarker for COVID-19 and as such has been included among candidate pharmacological targets for combating the severe forms of the disease. The use of VEGF-A inhibitors such as bevacizumab in critically ill patients is currently being investigated in clinical trials with encouraging results [105,123]. In parallel, work is underway to identify potential compounds that prevent SARS-CoV-2 from binding to NRP-1 [28,124,125].

## 5. Disruption of the NRP-1/VEGF-A Pathway by SARS-CoV-2 in Long COVID-19

Long COVID-19 is a recently described syndrome characterized by multiple organ symptoms that may occur in more than 50% of individuals infected with SARS-CoV-2 four weeks after recovery from acute illness [19,126,127,128]. Fatigue, cardiac arrhythmias, muscle weakness, impaired ability to perform activities of daily life, and neuropsychiatric disorders are among the most common manifestations [18,19]. The duration of symptoms varies widely, ranging from three weeks to more than three months according to various reports [129]. There is still uncertainty about the pathogenic mechanisms underlying the development of long COVID-19. Female gender and previous hospitalization due to severe illness are considered important risk factors. However, long COVID-19 can also occur in individuals with mild symptoms or without symptoms [129]. Sahanic et al. divided the post-COVID-19 sequelae into three phenotypes (hyposmia/anosmia phenotype, fatigue phenotype, and multi-organ phenotype), supporting the hypothesis that different pathogenic mechanisms may trigger the disease [128]. The clinical manifestations of long COVID-19 can be mild or severe and are likely due to both the cytopathic effects caused by the virus and the hyperactivation of the immune system. Patients with long COVID-19 may have impaired clearance of SARS-CoV-2, leading to a dysregulated immune response, which is in turn associated with increased thromboembolic risk [129]. In this context, neuropsychiatric and cardiovascular symptoms may be either due to organic injury (thromboembolism, local inflammation, fibrofatty substitution) or endothelial dysfunction and virus-induced demodulation of signaling pathways [9,126].

Very limited evidence links long COVID-19 to dysregulation of the NRP-1/VEGF-A pathway. An isolated cohort observational study of 103 SARS-CoV-2-infected patients, 48 of whom developed long COVID-19, reported significantly increased serum concentrations of VEGF in long COVID-19 patients compared with fully recovered subjects [130]. Three months after being discharged or onset of symptoms, patients with long COVID-19 also had higher anti-SARS-CoV-2 IgG titers and serum levels of granulocyte–macrophage colony-stimulating factor (GM-CSF). However, VEGF proved to be the only biomarker for long COVID-19 in univariate analysis. The study by Lim et al. also showed an increase in VEGF-A plasma levels in early (≤28 days) and late (>28 days) convalescent patients [102]. The authors also found that plasma levels of VEGF-A correlated directly with the severity of convalescence and circulating HLA-DR+CD38+CD8+ memory T lymphocytes and were instead negatively associated with CD8+CD56- mucosal-associated invariant T (MAIT) cells. HLA-DR+CD38+CD8+ T cells, which are typically elevated in severe forms of COVID-19 [131,132,133], reflect an exhausted T cell phenotype, whereas MAIT cells play a critical role in immune surveillance against pathogens [134]. Taken together, these data suggest that patients with worse COVID-19 outcomes have an altered immune profile with impaired clearance of SARS-CoV-2, which may accelerate inflammation and facilitate VEGF-A release. Indeed, epidemiological data show that long COVID-19 is more likely to follow severe forms of acute illness [129]. In the lung, the release of proinflammatory cytokines such as IL-1, IL-6, and TNF-α could stimulate the secretion of VEGF-A from alveolar epithelial cells and enhance lung inflammation [135]. However, in a Turkish study of 99 post-acute COVID-19 patients (who had been recovering for 3–12 weeks), the authors observed a significant decrease in serum concentration of VEGF-A in patients with evidence of pulmonary fibrosis on computed tomography (CT) of the chest compared with patients without pulmonary sequelae or healthy controls [136]. Thus, although VEGF-A appears to be a critical mediator of alveolocapillary permeability in ARDS, it may not be equally important in pulmonary fibrosis.

On the other hand, the results from other studies indicate that VEGF levels may be downregulated rather than increased during COVID-19 convalescence. A cross-sectional study on 20 healthy blood donors without previous SARS-CoV-2 infection and 140 convalescent plasma donors (median time since SARS-CoV-2 molecular positivity: 44 days) reported higher plasma levels of some cytokines, such as IFN-γ and IL-10, and lower plasma levels of VEGF-A in previously infected individuals compared to controls [137]. In another study, VEGF serum levels were reported to decrease in convalescent patients (time since symptoms: 35.75 ± 5.68 days) compared with the acute phase of disease and to be lower in asymptomatic SARS-CoV-2-infected individuals than in symptomatic patients [138]. Hence, the disturbance in the NRP-1/VEGF-A system by SARS-CoV-2 during long COVID-19 appears still uncertain. The conflicting results of the cited studies may depend on different methodologies, symptom persistence, concomitant cytokine milieu, viral clearance capacity, formation of neutralizing antibodies against the S protein, and medications.

Evidence of the dysregulation of VEGF-A-related signaling pathways during both acute and long COVID-19 is summarized in Table 3.

## 6. Disruption of the NRP-1/VEGF-A Pathway by COVID-19 Vaccines

COVID-19 vaccines have been rapidly developed using various technologies to counteract the global spread of infection. Currently approved formulations include nucleic acid-based vaccines, vector-based vaccines, recombinant protein vaccines, and inactivated SARS-CoV-2 vaccines. Although they have different pharmacological properties, they all encode or carry the SARS-CoV-2 S protein, which can elicit a selective immune response [139]. As of October 2022, more than 900 million doses of COVID-19 vaccines have been administered to people in the EU and the European Economic Area. Since their launch, the European Medicines Agency (EMA) has periodically been confirming the efficacy and safety profile of COVID-19 vaccines as determined in clinical trials, with serious adverse events considered extremely rare [140]. According to the most recent EudraVigilance report, adverse events are more common in women aged 18–64 years and include, in particular, constitutional, neurological, and musculoskeletal symptoms [20]. Serious cardiovascular events have also been described in rare cases. These data are consistent with Vaccine Adverse Event Reporting System (VAERS) reports and the published literature [21,22]. Occasionally, adverse events mimicking long COVID-19 manifestations have been reported, as shown in Table 4 [141,142,143,144].

Excessive reactogenicity to vaccine components or autoimmune phenomena have been proposed as possible mechanisms to explain neurological and cardiovascular side effects [145]. However, it has not been investigated whether the dysfunction of the NRP-1/VEGF-A system caused by the SARS-CoV-2 S protein may be directly associated with adverse events in COVID-19 vaccine recipients. A recent study has characterized the immune profile of six COVID-19-vaccinated male individuals, among whom there was one case of myopericarditis, and five male controls. Blood samples were collected 2–4 days after vaccination. By using a multiplex cytokine assay, the researchers reported a set of dysregulated cytokines and angiogenic mediators in recipients of COVID-19 vaccine versus healthy controls, including VEGF-A [146]. Remarkably, plasma concentrations of VEGF-A were lower in the vaccinated patient developing myopericarditis compared to the group of vaccinated individuals without any complications. Hence, even though preclinical evidence linked VEGF-A to the potential risk of some cardiac complications, such as arrhythmia [120], the real role of this mediator in post-vaccine cardiomyopathy remains undetermined. Interestingly, a recent Israeli cohort study examined 951 patients infected with SARS-CoV-2 and found that post-acute COVID-19 symptoms (fatigue, headache, limb weakness, and muscle pain) were lower in the vaccinated compared to the unvaccinated individuals [147]. These results could be due to a lower cytopathic effect of the virus or a more limited inflammatory response during infection in vaccinated subjects compared with unvaccinated subjects, although they could also reflect the antinociceptive function of the S protein [27].

Although current knowledge on circulating VEGF-A levels in COVID-19 vaccine recipients is limited to one study [146], the RS3PE syndrome has been described in two case reports as a consequence of vaccination, suggesting that an increase in this mediator may occur in a proportion of vaccinated individuals [148,149].

A number of studies have also demonstrated an association between COVID-19 vaccines and the risk of small fiber neuropathy (SFN) [23,24,25,26]. This is a clinical entity characterized by quantitative or qualitative damage to the small somatic and autonomic Aδ and C fibers, manifested by spontaneous pain, allodynia, hyperesthesia, or various autonomic dysfunctions [150]. In 50% of cases, the underlying cause is unknown and the disease is referred to as idiopathic; in the remaining cases, it may be the result of diabetes and other medical conditions. The occurrence of SFN has also been observed in SARS-CoV-2-infected patients, in whom it may accompany or follow acute symptoms of COVID-19 [151,152,153,154,155]. SARS-CoV-2 infection and COVID-19 vaccines may induce SFN via an immunological mechanism involving the proliferation of autoreactive T and B cells during seroconversion. Indeed, most reports describe the onset of SFN weeks after SARS-CoV-2 infection or vaccination, consistent with the activation of the adaptive immune response. Symptoms occurring within a week have been attributed to unmasking or flare-up of preexisting autoimmune or neurological diseases [24,155]. Conversely, there is still uncertainty about the role of the NRP-1/VEGF-A complex in the development of SFN. A study of skin punch biopsies from patients with diabetes-related SFN reported that the altered epidermal expression of NRP-1 may contribute to neuropathic pain and other neurological symptoms via the semaphorin-3A-mediated inhibition of C fiber sprouting and regeneration [39]. Other studies in diabetic animal models suggest that the *VEGF* gene transfer may improve SFN by increasing vascularization of nerves via the vasa nervorum [156]. The interplay of NRP-1, VEGF-Rs, and VEGF-A in nerves and blood vessels makes the association between SFN developing after COVID-19 or COVID-19 vaccination and increased VEGF-A levels plausible, but further research is needed.

## 7. SARS-CoV-2 S Protein Impairing the NRP-1/VEGF-A Pathway: A Potential Alternative Hypothesis Unifying Multiple Scenarios?

Although confirmatory data are lacking, some lines of evidence may suggest that cardiovascular and neurological manifestations developing in long COVID-19 patients and a proportion of COVID-19 vaccinated individuals could potentially depend on the pathogenic effects of the S protein in the neurovascular unit. Due to its dual binding to ACE2 and NRP-1, the SARS-CoV-2 S protein may cause the disruption of the corresponding signaling pathways with important effects on angiogenesis and nerve function. Such a “spike effect” has already been postulated with respect to the binding of ACE2. Indeed, studies have shown that the S protein may counteract the catalytic activity of the ACE2 receptor, leading to an imbalance in the formation of angiotensin II and angiotensin_1–7_ forms [30]. Given the relatively low abundance of ACE2 expression in the nervous cells and endothelial cells, an alternative scenario may be considered [107,116].

Specifically, it may be hypothesized that the SARS-CoV-2 S protein may impair NRP-1 molecular rearrangements that are necessary to the interaction with VEGF-A and VEGF-Rs, contributing to the increase in unbound (free) forms of VEGF-A observed during acute and long COVID-19 [101,102,130], which may be diverted to alternative pathways. For example, VEGF-A may bind to VEGF-R1 on immune cells and fuel inflammation [157]. Moreover, experiments in human breast cancer cells have shown that ACE2 downregulates the expression of VEGF-A and prevents the subsequent phosphorylation of extracellular signal-regulated kinases 1/2, which depends on the binding of VEGF-R2 [158]. Therefore, it may be expected that the S protein, once bound to the ACE2 receptor, may interfere with the control of ACE2 over VEGF-A synthesis, leading to a further increase in the levels of this mediator. The molecular homology between the S protein and VEGF-A could displace the latter from the binding site of NRP-1 [8], contributing to an increase in its serum concentration.

Moreover, proinflammatory cytokines synthesized during COVID-19 or in response to vaccines may promote the expression of VEGF-A [42,135], whereas inflammation and hypoxia may decrease the expression of VEGF-R2, NRP-1, and NRP-2, as observed in preclinical studies [159,160]. As a result, the release of VEGF-A may be uncoupled from the parallel upregulation of these receptors, with the consequence that the mediator would be redirected to bind VEGF-R1 on vessels and immune cells or VEGF soluble receptors. 

**Figure 3:**
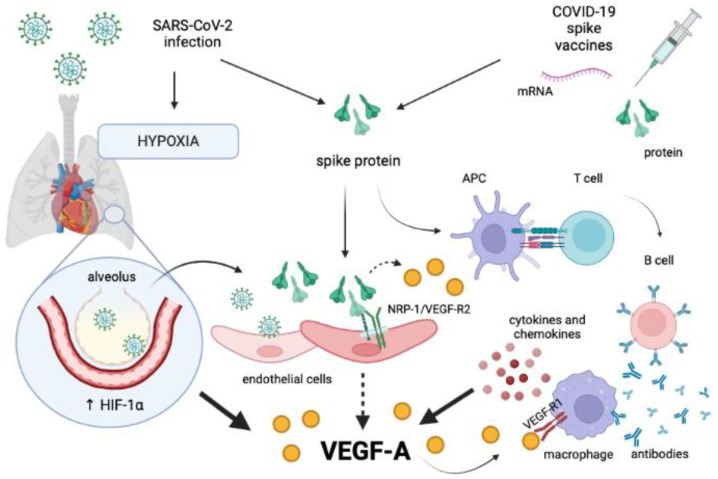
Hypothetical cascade of events triggered by SARS-CoV-2 S protein leading to overproduction of VEGF-A. The S protein produced by the virus during infection or used for vaccine immunization may induce the upregulation of VEGF-A in several ways. SARS-CoV-2 infection of pulmonary alveoli may result in hypoxia and local hyperexpression of HIF-1α, which is a potent trigger for VEGF-A release. Furthermore, during COVID-19 and following vaccination, the S protein can activate both the innate and acquired immune response, with the final secretion of cytokines from monocyte/macrophages. This event may be enhanced by the binding of anti-spike antibodies to the FcRs of these cells. The release of proinflammatory cytokines may further contribute to the increase in VEGF-A levels. Additionally, it may be supposed that the S protein may bind to NRP-1 in endothelial cells and destabilize the NRP-1/VEGF-R2 receptor complex, eventually preventing the docking to the endogenous ligand VEGF-A. Unbounded VEGF-A could be diverted to VEGF-R1 expressed on immune cells, amplifying the inflammatory background. Abbreviations: APC: antigen-presenting cell; COVID-19: coronavirus disease-19; HIF-1α: hypoxia-inducible factor 1α; NRP-1: neuropilin-1; VEGF-A: vascular endothelial growth factor A; VEGF-R1: vascular endothelial growth factor receptor 1; VEGF-R2: vascular endothelial growth factor receptor 2. Created with BioRender.com.

As for other viral infections, COVID-19 is characterized by a profound alteration of the immunological scenario. VEGF-A variations may therefore reflect changes in the levels of circulating proinflammatory mediators, such as TNF-α, IL-1β, IL-6, IL-8, MCP-1, and IFN-γ-inducible protein 10 (IP-10) [102,103,121,122,138]. Despite the conflicting data [102,137,138], such an immune dysregulation may persist in the convalescence period, contributing to alter the VEGF-A pathway. This cascade of events may be amplified by anti-SARS-CoV-2 S antibodies produced as the result of SARS-CoV-2 infection or vaccination. These antibodies may in fact activate monocyte/macrophage cells by interacting with fragment crystallizable receptors (FcRs) and stimulate the further release of proinflammatory mediators [161], as seen in Figure 3.

If these considerations prove to be true, the measurement of circulating VEGF-A could be an appropriate and noninvasive method to assess the occurrence of neurovascular dysfunction in long COVID-19 subjects or vaccinated individuals with cardiovascular or neurological symptoms not otherwise explained by instrumental tests. At the same time, drugs that can inhibit VEGF-A may be considered for the treatment of persistent or severe manifestations that do not respond to conventional treatments. However, the existence of different VEGF-A isoforms, the bioavailability of VEGF-A, and laboratory methodology are major limitations of this approach. As mentioned previously, VEGF-A can be spliced into different variants that have opposite effects and may be collectively involved in increasing the total VEGF-A concentration [42,43,44]. The abundant release of VEGF-Axxxb isoforms may reduce the efficacy of anti-VEGF-A agents such as bevacizumab due to the nonspecific binding of the drug to unintended VEGF-A splice variants [162]. Unfortunately, studies examining concentrations of circulating VEGF-A in acute or post-COVID-19 patients have not distinguished between the different VEGF-A isoforms [101,102,130,136], so the true contribution of each splice variant to clinical manifestations is unknown. Furthermore, there is heterogeneity in the choice of samples (plasma or serum) used to measure VEGF-A in these patients. It is reasonably well known that the concentration of VEGF-A in serum samples may be affected by the number of platelets, because platelets are a source of VEGF-A that could be released during clotting events for serum separation. Therefore, searching for VEGF-A in plasma samples could more accurately reflect the actual concentrations of this mediator in peripheral blood [163]. Finally, the level of circulating VEGF-A may depend on the amount of VEGF-A scavenger receptors, which could reduce the availability of VEGF-A in peripheral blood [101].

## 8. Conclusions

COVID-19 is a complex disease whose pathogenesis, clinical course, and potential pharmacological approaches have been extensively studied, resulting in an unprecedented amount of scientific knowledge accumulated in just over two years. Recent research suggests that COVID-19 is the result of a profound endotheliopathy caused by both direct viral damage and hyperactivation of the immune response. In addition to favoring virus entry, the SARS-CoV-2 S protein may interfere with the physiological function of bound receptors, giving rise to dysfunctional complications. The impairment of ACE2 receptor activity has already been deciphered in relation to COVID-19 cardiovascular manifestations. Recently, a potential dysfunction of the NRP-1/VEGF-A complex in acute COVID-19 has begun to be unveiled. However, the contribution of this process to the development of long COVID-19 or certain side effects of COVID-19 vaccines is still unclear. It may be possible that the SARS-CoV-2 S protein produced by the virus or used for immunization could interfere with NRP-1/VEGF-A-mediated pathways, altering their physiological processes and thereby causing symptoms even in the absence of organic damage. Although evidence in favor of this is still scarce, the levels of circulating VEGF-A may be elevated in long COVID-19 patients and vaccinated individuals suffering from certain side effects, suggesting that VEGF-A could be one of the possible biomarkers for monitoring such neurovascular defects [105,106,117]. However, given the lack of experimental data, further studies are indeed needed to verify this theory.

## Figures and Tables

**Table 1:** Summary of the clinical studies linking VEGF dysregulation to human respiratory viral infections.

Author, Year	Virus	Type of Study	Population Studied	Main Results
Morichi et al., 2017 [75]	Influenza	Case control study	-11 pts with IAE -6 pts with BM -24 pts with non-central nervous system infection	Increased levels of VEGF and PDGF in cerebrospinal fluid of IAE and BM pts compared with controls; positive correlation between VEGF and PDGF cerebrospinal fluid levels and worse prognosis according to the PCPC score
Wong et al., 2018 [77]	Influenza	Case control study	-27 pts with SARI -27 non-hospitalized pts with ILI	Higher VEGF-A serum levels during both acute disease and convalescence (2 weeks later) in pts with SARI compared to those with ILI
Bautista et al., 2013 [78]	Influenza	Case control study	-32 pts with A/H1N1 virus infection (17 with ARDS-related AKI and 15 ARDS pts without AKI) -18 HCs	Higher serum levels of VEGF in ARDS/AKI pts; significant association between VEGF serum levels and the risk of AKI development in ARDS pts; acute tubular damage and diffuse expression of VEGF in the cytoplasm of renal tubules with hydropic degeneration observed at immunohistochemistry assays in ARDS/AKI pts with fatal outcome
Pino et al., 2009 [79]	RSV	Observational, longitudinal study	-20 hospitalized children due to severe infection -12 HCs	Hypersecretion of VEGF in NPAs from hospitalized children at discharge and 1 yr later; potential role of VEGF in contributing to the pathogenesis of post-infectious wheezing
Bermejo-Martin et al., 2007 [80]	RSV	Case control study	-22 children aged <2 yrs with severe infection involving the lower respiratory tract -22 children with innocent heart murmurs	Positive correlation between plasma levels of VEGF and disease severity
Lee et al., 2000 [83]	RSV	Prospective study	-25 pts with documented influenza infection -47 pts with documented RSV infection -21 controls without documentable virus infection	Higher levels of VEGF in NPA samples from RSV-infected pts compared to the other groups; detection of VEGF 165– and 121–amino acid isoforms as the major VEGF representatives in nasal secretions of RSV-infected individuals

Abbreviations: AKI: acute kidney injury; ARDS: acute respiratory distress syndrome; BM: bacterial meningitis; HCs: healthy controls; IAE: influenza-associated encephalopathy; ILI: influenza-like illness; NPAs: nasopharyngeal aspirates; PCPC: pediatric cerebral performance categories; PDGF: platelet-derived growth factor; pts: patients; RSV: respiratory syncytial virus; SARI: severe acute respiratory illness; VEGF: vascular endothelial growth factor; yr: year.

**Table 2:** Amino acid sequences of VEGF members and isoforms and SARS-CoV-2 S1 subdomain predicted to bind to the B1 domain of NRPs. Abbreviations: NRP: neuropilin; VEGF: vascular endothelial growth factor.

NRP Ligand	Binding Sequence (C-Terminus)
VEGF-A165	DKP**RR**
VEGF-B167	**R**KL**RR**
VEGF-B186	**R**PQP**R**
VEGF-C	SII**RR**
spike S1 subdomain	TNSP**RR**A**R**

**Table 3:** Summary of the studies reporting altered VEGF-A expression in acute and long COVID-19/convalescent patients or suggesting the efficacy of anti-VEGF-A interventions in acute disease.

Author, Year	Type of Study	Population Studied	Main Results
Rovas et al., 2021 [101]	Prospective, observational, cross-sectional study	-23 pts with moderate–severe COVID-19 -15 HCs	Increase in plasma VEGF-A levels and significant correlation with 60-day in-hospital mortality
Lim et al., 2021 [102]	Cross-sectional study	-37 pts with acute COVID-19 -40 convalescent pts -10 HCs	Increase in VEGF-A plasma levels during both acute disease and convalescence; positive correlation between VEGF-A plasma levels and convalescence severity; inverse correlation between VEGF-A plasma levels and CD8+CD56- MAIT cells during convalescence; positive association between VEGF-A plasma levels and HLA-DR+CD38+ CD8+ T cells in convalescent pts
Medeiros et al., 2022 [103]	Longitudinal study	-82 hospitalized moderate-to-severe COVID-19 pts, 41.5% of whom developing AKI	Increased serum levels of VEGF in pts with COVID-19-related AKI compared to pts without renal involvement
Choreño-Parra et al., 2021 [121]	Prospective cohort study	-10 pts with acute moderate COVID-19 -24 pts with acute severe COVID-19 -23 pts with pandemic influenza A(H1N1)	Increased serum levels of VEGF along with systemic, Th1 and Th2 cell cytokines in pts with COVID-19 but not in those with influenza
Zhang et al., 2022 [122]	Case control study	-20 hospitalized COVID-19 children -58 children with ARTI caused by RSV, influenza virus, and ADV -20 HCs	Significantly increased VEGF serum levels in SARS-CoV-2-infected pts compared to the other groups
Pang et al., 2021 [123]	Single-arm trial	-26 pts with severe COVID-19 treated with a single dose of bevacizumab	Improvement in PaO2/FiO2 parameters at days 1 and 7 from baseline; improvement in oxygen-support status in 92% of pts at day 28 from baseline; decrease in lung lesions on chest CT or X-ray within 7 days; normalization of body temperature within 72 h in 93% treated pts
Torres-Ruiz et al., 2021 [130]	Observational cohort study	-103 pts with previous COVID-19, 46.6% of whom diagnosed with long COVID-19	Higher VEGF serum levels in pts with long COVID-19 than in pts without; VEGF appearing as the sole biomarker strongly associated with long COVID-19 in univariate analysis
Arslan et al., 2022 [136]	Observational, cross-sectional study	-32 pts with a previous COVID-19 diagnosis and no lung fibrosis on CT scan -32 pts with a previous COVID-19 diagnosis and lung fibrosis on CT scan -26 HCs	Higher VEGF serum concentration in pts with a previous COVID-19 diagnosis and no lung fibrosis compared to the other groups
Bonny et al., 2021 [137]	Observational, cross-sectional study	-20 healthy blood donors without previous SARS-CoV-2 infection -140 COVID-19 convalescent plasma donors	Higher plasma levels of IFN-γ, IL-10, IL-15, IL-21, and MIP-1 and lower levels of IL-1RA, IL-8, IL-16, and VEGF-A in convalescent plasma donors compared to controls
Chi et al., 2020 [138]	Observational, cross-sectional study	-70 SARS-CoV-2-infected pts (4 asymptomatic; 66 symptomatic) -4 convalescent pts -4 HCs	Higher VEGF serum levels in symptomatic cases compared with asymptomatic cases; lower serum levels of VEGF in convalescent pts than in symptomatic pts; positive correlation between VEGF serum concentration and male gender; weakly positive correlation between VEGF serum concentration and SARS-CoV-2 viral load

Abbreviations: ADV: adenovirus; AKI: acute kidney injury; ARDS: acute respiratory distress syndrome; ARTI: acute respiratory tract infection; COVID-19: coronavirus disease-19; CT: computed tomography; IL: interleukin; IL-1RA: IL-1 receptor antagonist; IFN: interferon; HCs: healthy controls; HLA: human leukocyte antigen; MAIT: mucosal-associated invariant T; MIP-1: macrophage-inflammatory protein-1; PaO2/FiO2: ratio of arterial oxygen partial pressure (PaO2 in mmHg) to fractional inspired oxygen; RSV: respiratory syncytial virus; pts: patients; SARS-CoV-2: severe acute respiratory syndrome coronavirus-2; VEGF-A: vascular growth factor-A.

**Table 4:** Constitutional, musculoskeletal, neuropsychiatric, and cardiovascular manifestations reported during long COVID-19 or listed among the COVID-19 vaccine side effects.

Clinical Domain	Long COVID-19	COVID-19 Vaccine Side Effects
Constitutional	Fatigue Sleep disturbances	Fever Fatigue
Musculoskeletal	Arthralgias Myalgias Arthritis	Arthralgias Myalgias
Neuropsychiatric	Paresthesia Hypoesthesia Dysesthesia Headache Dizziness SFN PTSD Anxiety Depression Emotional disturbances Brain fog Hyposmia Hypogeusia	Weakness Headache Dizziness Transient sensory symptoms SFN Seizures Guillain–Barré syndrome Transverse myelitis Encephalopathy Cerebral vascular events Psychosis and confusional states New-onset bipolar disorder Anxiety
Cardiovascular	Palpitations Chest pain Dyspnea Increased CV disease risk	Syncope Palpitations Myocarditis Pericarditis Thrombosis

Abbreviations: COVID-19: coronavirus disease-19; CV: cardiovascular; PTSD: post-traumatic stress disorder; SFN: small fiber neuropathy.

## Data Availability

Not applicable.
